# GIS and cellular automata based slope rainwater movement process model and its application

**DOI:** 10.1038/s41598-024-60263-8

**Published:** 2024-04-28

**Authors:** Lei Liu, Yu Chen, Yanjun Zhang, Zhipeng Lian, Laizheng Pei, Yalei Liu

**Affiliations:** 1 Wuhan Center, China Geological Survey (Geosciences Innovation Center of Central South China), Wuhan, 430205 Hubei China; 2 Innovation Base for Geo-safety Risk Forecasting and Prevention Technology in Central South China, Geological Society of China, Wuhan, 430205 Hubei China

**Keywords:** Rainfall, Mountain slopes, Runoff movement, Infiltration model, Cellular automata, Rainwater movement, Environmental sciences, Hydrology, Natural hazards

## Abstract

Rainfall serves as a significant factor contributing to slope stability challenges in mountainous areas, and simulating the process of slope rainwater movement is a crucial approach for analyzing the stability of slopes triggered by rainfall. By combining computer numerical simulation technology with traditional hydraulic and hydrological calculation theories, it is possible to create an efficient and precise rainwater movement model that can simulate and analyze the process of rainwater movement on slopes. Utilizing natural slopes as the focal point of our research, the cellular automaton method was applied to simulate rainfall runoff on slopes, and a Cellular Automata (CA) based model for rainwater movement process was developed. This model modified the Green-Ampt (G-A) infiltration model by adopting an elliptical water content curve and introducing a coefficient that quantifies the ratio of saturated to unsaturated depth. Additionally, we refined the rules governing runoff generation and convergence within the slope and on its surface, enabling a comprehensive simulation of the entire rainwater movement process on the slope. Furthermore, the effectiveness of the model was validated through analytical solutions derived from simplified assumptions, laboratory experiments on infiltration and runoff in the flume, and a case study of a natural slope. The results show that the infiltration calculation results of the rainwater movement model are closer to the experimental values, and their overall values are slightly higher than the measured values, which are basically consistent with the model test results; The runoff calculation results show a phenomenon of initially increasing and gradually approaching the measured values compared to the measured values. When applying the model to an actual slope, it was found that the model comprehensively accounts for the influence of slope seepage, infiltration and runoff process, has better performance compared to G-A modified model. The model can be used to describe the spatial distribution and temporal variation of infiltration and runoff processes.

## Introduction

Rainfall significantly impacts slope stability in mountainous areas^1^.. From geomorphology, hydrogeology, and engineering geology perspectives, the underlying mechanism governing slope stability involves the transformation of rainwater into underground and surface water^2^. Slope instability can arise when rainwater infiltrates deep into the slope, ultimately leading to a reduction in the shear strength of the soil. Rainwater that does not infiltrated the interior of the slope collects on its surface, subsequently giving rise to overland flow, also known as runoff. The runoff can cause planar erosion, potentially triggering shallow flow on the surface of the slope ^3^. Therefore, paying attention to the dynamics of rainwater movement on slopes has become an important aspect in the study of slope stability.

The process of rainwater movement on slopes is complex. The lateral and vertical movement of precipitation, once it falls on the surface, are influenced by various factors such as land terrain, topography, vegetation, soil texture. The unsaturated and saturated zones of soil, along with surface water, govern hydrological processes including infiltration, evapotranspiration, and runoff^4^. Research in the literature on calculation models for unsaturated soil water infiltration depth mainly includes empirical formula methods such as Green-Ampt Model (G-A)^5^、Kostiakov Model^6^、Horton Model^7^、Philip Model ^8^, et al., and Richard differential equation method. The Richard method, with its integration of Darcy's law and the continuity equation, offers a highly accurate computational approach to studying the rainwater movement in variably saturated soil. However, in engineering practice, this approach encounters challenges such as the difficulty in determining boundary conditions, as well as the intricacies with its complex differential equations and numerical solution process^9^. In recent years, the G-A model, based on capillary theory, has garnered widespread attention compared with other empirical formula methods. This is primarily due to its concise and straightforward physical interpretation, accessible calculation parameters, and convenient application in engineering practice^10–14^. While previous research results have significantly broadened the applicability of the model, they have overlooked the objective fact of the existence of unsaturated zone.

As rainfall infiltration continues, ponding may occur on the surface, leading to runoff along the slope. Over the past half-century, the continuous deepening of research on various elements involved in rainwater movement, coupled with the rapid development and application of technologies such as computers and Geographic Information Systems (GIS), has led to the gradual emergence of two categories of physical-based hydrological and hydrodynamic models, which have been increasingly utilized to calculate runoff processes^15^. Hydrodynamic methods, such as two-dimensional hydraulic models, can obtain relatively accurate simulation results but require a considerable quantity of input parameters, a complex modelling process, and solving tedious differential equations, which have limited Its practicality^16^. Distributed hydrological models, including IHDM^17^、MIKE SHE^18^、TOPMODEL^19^、Arc Hydro Data Model^20^、SWAT^21^、HEC-HMS^22^, primarily focus on watersheds. These models divide the surface and underground systems into distinct regions and couple the control equations that describe various processes within each region using iterative solving methods. However, their application accuracy is relatively low when it comes to the slope scale. Overall, these models are primarily simulated on small scales, using watersheds as the basic hydrological units, which precludes them from accurately reflecting interactions among watersheds, and makes it difficult to perform precise spatial analysis within the watersheds. As a result, the research findings of the watershed scale are challenging to extrapolate to larger scale, particularly the slope scale. In recent years, the development of rainfall-runoff models utilizing Cellular Automata (CA) have demonstrated to be relatively simple compared to hydrodynamic models, while maintaining sufficient computational accuracy. These models possess the capability to not only calculate the runoff and infiltration of slopes during rainfall events but also dynamically visualize the spatial variation of the calculated results, which offers significant application prospects^23–27^.

This research focuses on natural slopes and utilizes applies cellular automata method to simulate the rainwater movement on slopes, developing a CA-based model for the rainwater movement process. The model modifies the G-A infiltration model by adopting an elliptical water content curve and introducing coefficients to quantify the ratio of saturated and unsaturated depths. Additionally, the model improves the runoff generation rules on the slope and seepage within the slope , enabling the simulation of the entire rainwater movement process across the slope. Furthermore, the effectiveness of the model was validated through analytical solutions derived from simplified assumptions, laboratory experiments on infiltration and runoff in the flume, and a case study of a natural slope.

## GIS based slope rainwater movement process model

### Ideology

**Figure 1:**
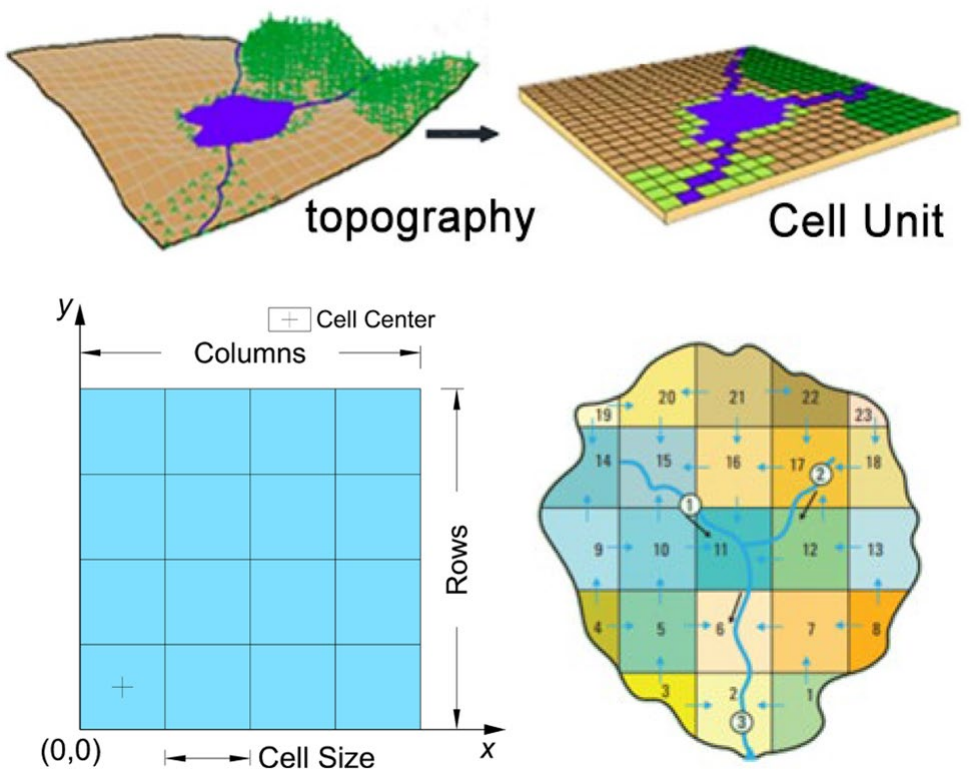
Schematic diagram of surface information represented by raster data.

**Figure 2:**
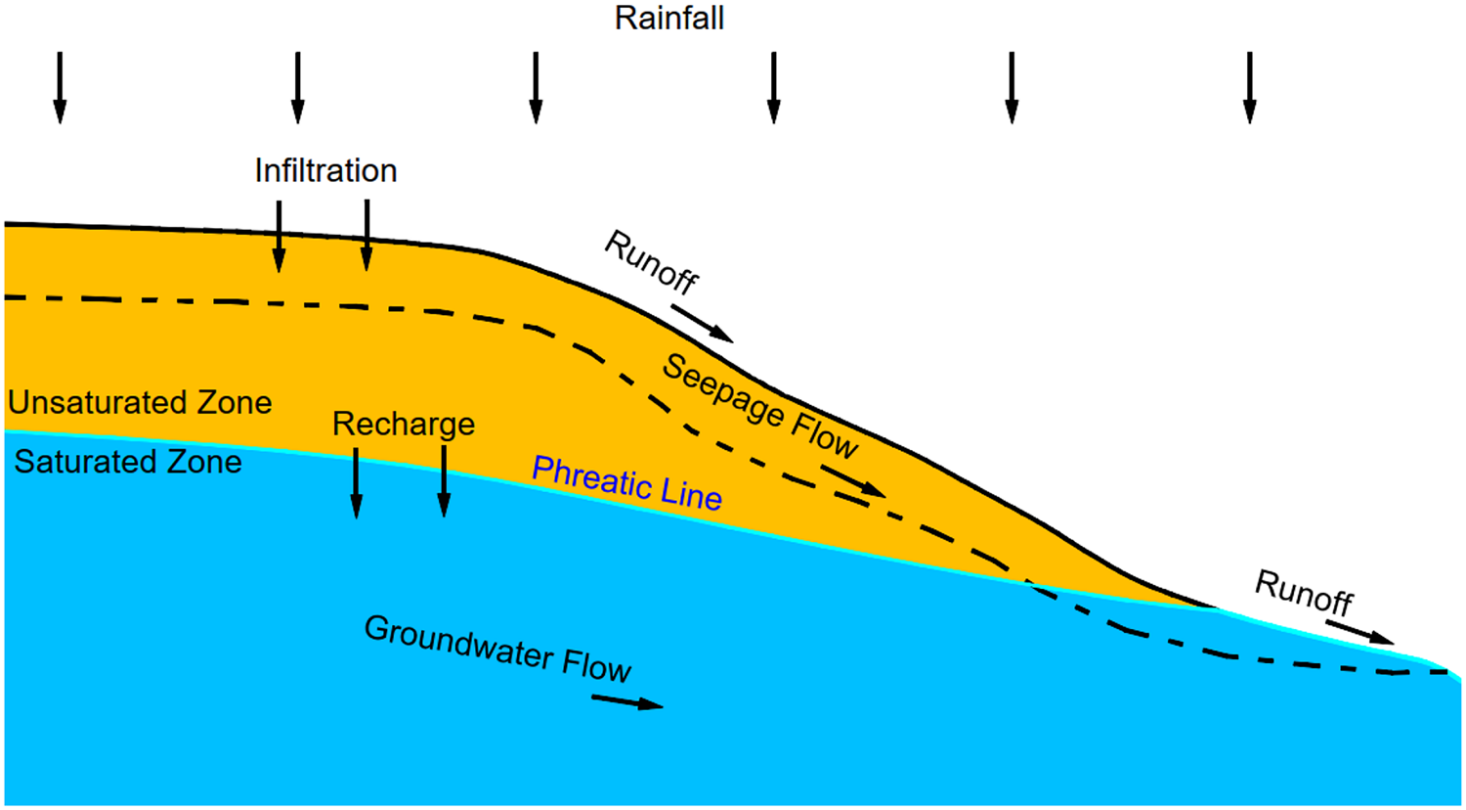
Schematic diagram of rainfall movement process on a slope.

The various types of information about real slopes, such as elevation, slope, groundwater level, etc., display distinct spatial distribution characteristics. They can be projected onto a plane and represented using uniformly divided grids, which are stored in the form of raster data in GIS. Each grid represents a property value, as shown in Fig. 1. Therefore, all data describing real slope rainwater movement can be abstracted as the raster layer of GIS. Rainwater movement across slopes, encompassing both infiltration and runoff, is characterized by continuous spatiotemporal patterns. Rasterized data subdivides the continuity of spatiotemporal data, reflected in the movement of rainwater from one grid to another within specific time intervals. Specifically, during a given rainfall process, at each calculation step, it is necessary to determine whether the grid cells representing the slope surface has ponding. The absence of ponding indicates that there has been no runoff at this moment, and it is considered that all rainfall has fully infiltrated into the slope. The rainfall infiltration amount of each grid is calculated, and then continuing to the next step calculation. Conversely, ponding signifies the occurrence of runoff at the grid at this time step. Calculate the water depth on the slope surface and saturated aquifer depth inside the slope of the grid currently. Based on the terrain characteristics, calculate the runoff on the slope surface and seepage inside the slope, update each grid's ponding depth and infiltration depth. Repeat the iterative calculation until the final time is completed. The schematic diagram of rainfall movement process on a slope is shown in Fig. 2.

### Cellular automata

Cellular Automata is a dynamic simulation system with discrete spatiotemporal and state variables that continuously evolve according to established rules, characterized by local interactions, self-updating, and universal computing. The CA model is described statistically as follows:

1 $$CA = \left\langle {L_{d} ,N,S,f} \right\rangle$$

where, L represents the cellular space, d represents the dimension of the cellular space, N represents the set of all cellular neighbor around the cell; S represents the set of cellular states; F represents the conversion rule corresponding to the cell.

**Figure 3:**
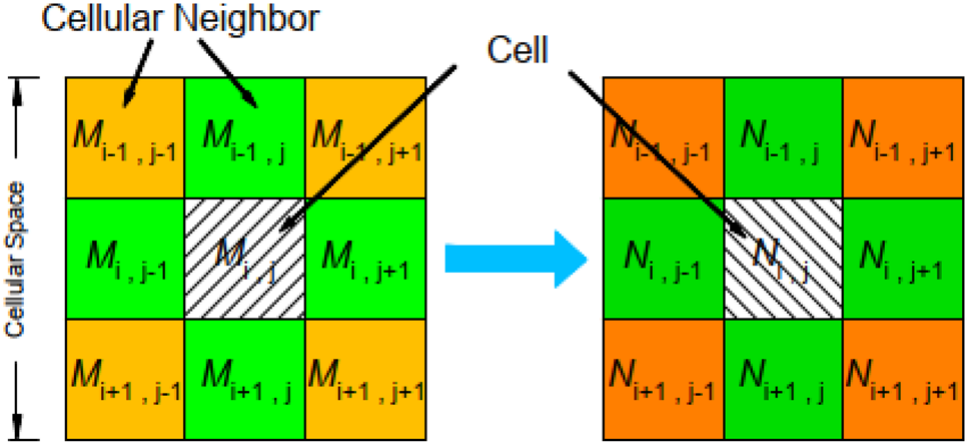
Schematic diagram of cellular automaton model composition.

CA divides the system into regular cellular grids, each of which is in a limited state and influenced by adjacent cellular states. During each simulation step, cellular states are synchronously updated according to specified transition rules to simulate the complex nonlinear physical phenomena. A typical cellular automaton model consists of four parts: discrete space, neighborhood, cellular state, and transition rule (Fig. 3). This study uses the most common two-dimensional square cell and Moore neighborhood to simulate the process of rainwater movement.

### Cellular transition rules

Developing cellular transition rules is pivotal in constructing cellular automata models. This study decomposes the calculation of rainfall movement on slopes into two processes: infiltration and runoff, and constructs transition rules for each process. Infiltration rules account for infiltration factors such as rainfall, moisture content, and topography. The runoff rules mainly involve identifying the flow direction of water on the slope surface and inside the slope and allocating the flow reasonably.

#### Rainwater movement rules

The process of rainwater movement is the most basic hydrological process on slopes. Rainfall falls on the slope surface, and after being intercepted by plants, filled with depressions on the ground, soil infiltration, ground evaporation, and plant transpiration, the remaining precipitation then forms surface runoff. This process is influenced by many factors, such as climate factors, slope factors, human factors, etc. Climate factors mainly include rainfall, evapotranspiration, temperature, humidity, etc. Slope factors mainly include slope shape and area, terrain and landforms, hydrogeological characteristics, elevation, slope, slope orientation, etc. Human factors mainly refer to human activities, such as construction of various water conservancy projects, excessive logging of trees, land slope reclamation, disorderly exploitation of underground resources, etc. Water balance is the driving force behind the movement of rainwater within the slope. With the runoff generation law, a water balance equation can be established:

2 $$SW_{t} = SW_{0} + \sum\limits_{i = 1}^{t} {(q_{i} - Q_{surf} - E_{a} - Q_{infil} - Q_{gw} )}$$

where *SW*_*t*_ is the final soil water content, *SW*_0_ is the initial soil water content on day *i*, *t* is the time, *q*_*i*_ is the amount of precipitation on day *i*, *Q*_*surf*_ is the amount of surface runoff on day *i*, *E*_*a*_ is the amount of evapotranspiration on day *i*, *Q*_*infil*_ is the amount of water entering the vadose zone from the soil profile on day *i*, and *Q*_*gw*_ is the amount of return flow on day *i*.

#### Rainfall infiltration rule

Infiltration prediction directly determines the accuracy of the rainwater movement model, as it controls the quantity of water that penetrates the slope, and the amount that flows as runoff on the surface and as seepage within the slope. Several physically based and empirical infiltration models have been developed to quantitatively analyze the infiltration process. The study employs the G-A model due to its simplicity, good fit to data and the ability to obtain its parameter values.

The G-A model, proposed by Green and Ampt^5^, is a capillary theory-based physical model for soil infiltration. Characterized by its simple expression, minimal model parameters, and straightforward physical meaning, it has been widely used in the field of soil infiltration research.

The classic G-A model conceptualizes the process of water infiltration as a "piston-like" motion, where the wetting front serves as a distinct interface separating the dry and wet zones. The model represented as follow:

3 $$i_{w} = K_{s} \left( {\frac{{z_{w} + H + \psi_{w} }}{{z_{w} }}} \right)$$

where *t* represents a certain moment; *z* is the depth below the surface; *z*_*w*_ is the depth of the wetting front at time *t* (the subscript "*w*" indicates the location of the wetting front), *h*_0_ = *H* is the ponding head at the surface, *θ*_*s*_ is the saturated moisture content of the soil, *θ*_*i*_ is the initial moisture content of the soil, *K*_*s*_ is the saturated permeability coefficient of the soil; *ψ*_*w*_ is the average matric suction head of the wetting front.

##### Green-ampt model considering the influence of slope

**Figure 4:**
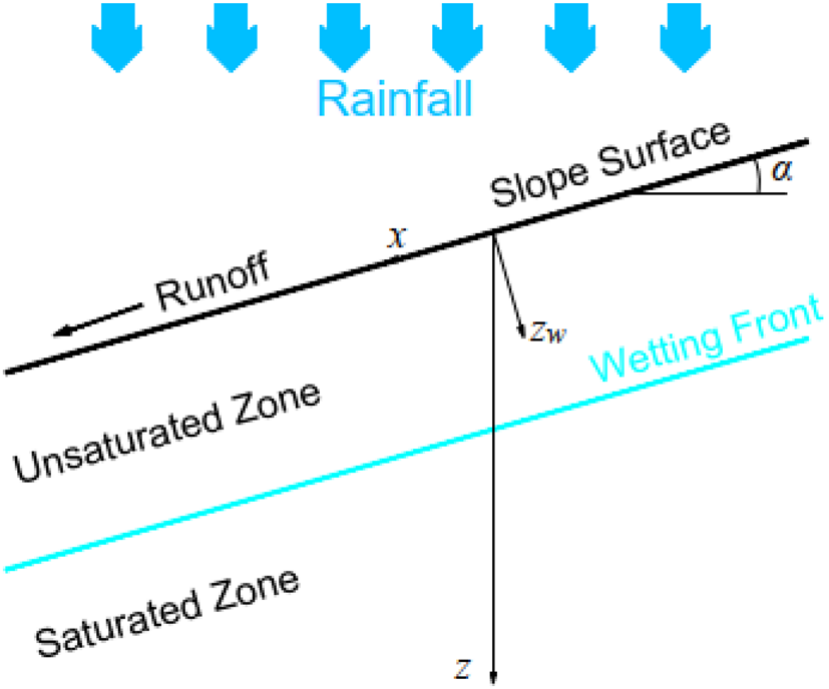
Green-Ampt Slope Infiltration Model.

Chen et al.^28^ extended the G-A model to investigate rainfall infiltration on infinite slopes (Fig. 4), assuming that the wetting front remains parallel to the slope surface. Given a slope angle of *α*, the G-A slope infiltration model can be expressed as follows,

4 $$i_{w} = K_{s} \left( {\frac{{z_{w} \cos \alpha + H + \psi_{w} }}{{z_{w} }}} \right)$$

Mein^29^ argued that surface ponding occurs when the rainfall intensity *q* exceeds the soil infiltration rate *i*. Denoting the time at which ponding begins as *t*_*p*_ and the corresponding depth reached by the wetting front as *z*_*p*_, the infiltration rate *i* at this point is *q*cos*α*. Substituting above into Eq. (3) yields:

5 $$z_{p} = \frac{{K_{s} \cdot \psi_{w} }}{{(q - K_{s} )\cos \alpha }};t_{p} = \tfrac{{(\theta_{s} - \theta_{0} )\psi_{w} }}{q\cos \alpha }$$

The accumulated infiltration amount before ponding is calculated as follows:

6 $$Q_{infil} = q\cos \alpha \cdot t$$

The accumulated infiltration amount after ponding is calculated as follows:

7 $$Q_{infil} = i_{w} \cdot t = K_{s} \left( {\frac{{z_{w} \cos \alpha + H + \psi_{w} }}{{z_{w} }}} \right) \cdot t$$

The relationship between infiltration depth *z*_*GA*_ and infiltration amount:

8 $$z_{GA} \cos \alpha = \frac{{Q_{infil} }}{{\theta_{s} - \theta_{0} }}$$

In summary, the relationship between infiltration depth and time in the slope can be calculated using the following formula:

9 $$\frac{{dz_{GA} }}{dt} = \left\{ {\begin{array}{*{20}c} {\frac{q\cos \alpha }{{\theta_{s} - \theta_{0} }}} & {t \le t_{p} } \\ {\frac{{K_{s} \left( {z_{GA} \cos \alpha + H + \psi_{w} } \right)}}{{z_{GA} \left( {\theta_{s} - \theta_{0} } \right)}}} & {t > t_{p} } \\ \end{array} } \right.$$

##### Modified green-ampt model

**Figure 5:**
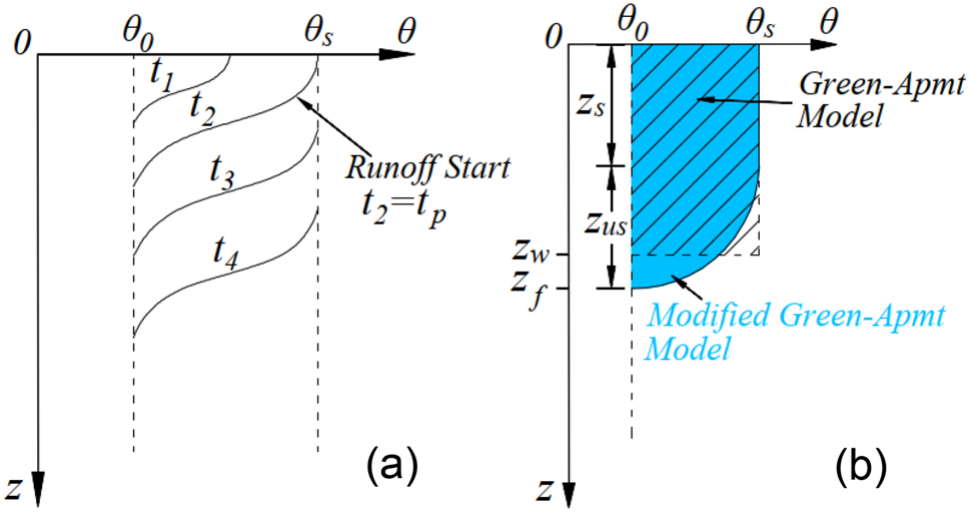
Schematic diagram of modified G-A model Parameter Determination.

The G-A model generalizes the moisture content curve shown in Fig. 5a into a rectangular shape, with some deviation from the actual infiltration test results^30^. Drawing upon the research findings of Peng^31^, this study modified the G-A model. Specifically, the moisture content curve after the start of ponding is approximated as a rectangular saturated zone with a length of *z*_*s*_ above, followed an elliptical unsaturated zone with a length of *z*_*us*_ below. The ratio of *z*_*us*_ to *z*_GA_ is *λ*, i.e. *z*_*s*_ = (1-*λ*)*z*_GA_. As shown in Fig. 5b, The modified infiltration depth *z*_*m*_ can be obtained as follows:

10 $$z_{m} = \frac{{(4 - \pi )b - 4 + \sqrt {(\pi b - 4b + 4)^{2} + 16a(4 - \pi )z_{GA} } }}{2a(\pi - 4)}$$

where, *a* and *b* are coefficients, and a < 0, 0 < b < 1, which can be obtained by fitting the data from indoor soil column experiments.

##### Parameter determination

Determining the saturated permeability coefficient *K*_*s*_ and matrix suction head *ψ*_*w*_ poses significant challenges in applying the Green Ampt model. The soil saturated hydraulic conductivity *K*_*s*_ is mainly affected by soil texture, structure, organic matter content, and bulk density. It can be measured through on-site methods such as single-ring infiltrometers, double-ring infiltrometers, and tension disc infiltrometers, or laboratory measurements such as the constant head method and falling head method^32^.

The average or effective matric suction head at the moist front *ψ*_*w*_, is generally believed that Mein^29^ proposed the use of soil water suction *ψ* Weighted average of *ψ*_*w*_, is more reasonable, it is a function of water content, which can be solved through experiments or the following equation:

11 $$\psi_{w} = \int_{0}^{{\psi_{i} }} {K_{r} \left( \psi \right)d\psi }$$

where, *ψ*_*i*_ is matrix suction head corresponding to the initial moisture content *θ*_*i*_, *K*_*r*_ is the relative permeability coefficient, *ψ* (*ψ* >  = 0) is matrix suction head corresponding to the moisture content *θ*.

The soil water characteristic curve (SWCC) and relative permeability coefficient *K*_*r*_ of soil can be solved as follows:

12 $$\theta (\psi ) = \theta_{r} + \frac{{\theta_{s} - \theta_{r} }}{{\left[ {1 + \left( {\alpha \psi } \right)^{n} } \right]^{m} }}$$

13 $$K_{r} (\psi ) = \frac{{\left\{ {1 - \left( {\alpha \psi } \right)^{n - 1} \left[ {1 + \left( {\alpha \psi } \right)^{n} } \right]^{ - m} } \right\}^{2} }}{{\left[ {1 + \left( {\alpha \psi } \right)^{n} } \right]^{m/2} }}$$

where, *θ*_*r*_ is the soil residual moisture content; α, n, m is the fitting parameter, in the VG-Mualem model, m = 1–1/n; In the VG-Burdine model, m = 1–2/n. In this paper, the matric suction at the wetting front is taken as half of the air entry value.

#### Runoff rule

Gravity causes the ponding to flow downhill, generating runoff. Acknowledging the intricacies of the runoff’s hydraulic conditions, this study simplifies the runoff process and applies the method of open-channel hydraulics for an approximate simulation.

##### Determination of water flow direction

**Figure 6:**
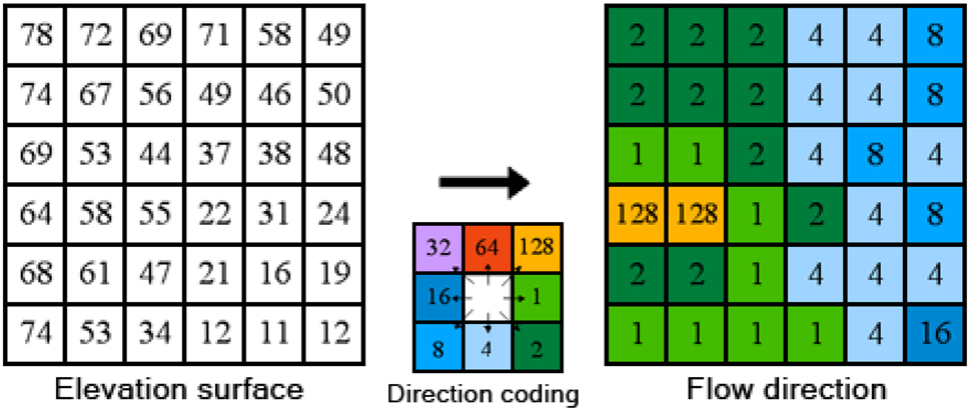
Schematic diagram of the eight flow model (D8) algorithm.

Employing the steepest slope algorithm, this study presumes that water in each cell consistently flows towards the lowest cell. This involves computing the slope between a cell and its neighboring cells, determined by dividing the cell height difference by the distance between cells. The neighboring cell with the steepest slope is designated as the outflow cell. Additionally, The eight flow direction model proposed by Jenson^33^ is utilized to represent the aspect, where each direction corresponds to a specific number (1: East; 2: Southeast; 4 South; 8: Southwest; 16: West; 32: Northwest; 64: North; 128: Northeast).The schematic diagram of the model is depicted in Fig. 6.

##### Slope surface runoff flow

Using the steepest slope algorithm, the central cell allocates surface water to the neighboring cells until an average water level is reached. However, during the actual slope runoff process, disparities in water level, roughness, and slopes across cells result in different flow velocities between them. Consequently, it is imperative to calculate the velocities and capacities of the flow within each cell.

The flow velocity *v*_*sf*_ of water on the slope of central cell is calculate using the Manning formula:

14 $$v_{sf} = \frac{1}{n}H^{\frac{2}{3}} J^{\frac{1}{2}}$$

where, *H* is the water depth of the central cell, in meters; *n* is the Manning coefficient; *J* is the slope.

Within the time step ∆*t*, the capacities of runoff on the slope of central cell can be expressed as:

15 $$Q_{out\_sf} = v_{sf} \cdot A_{sf} \cdot \Delta t = \frac{1}{n}H^{\frac{5}{3}} J^{\frac{1}{2}} \cdot d \cdot \Delta t$$

where, *d* is the cell width; *A*_*sf*_ is the cross-sectional area of the water flow on the slope of central cell.

##### Inside slope seepage flow

Controlled by the slope geometric features and hydraulic gradients, the water within the saturation zone of the central cell will be allocated to the neighboring cells under the seepage rules. According to Darcy's law, the flow velocity of water in the saturated zone of central cell, denoted as *v*_*s*_, can be calculated as follows:

16 $$v_{s} = K_{s} \cdot \frac{{z_{s} }}{L}$$

Within the time step ∆*t*, the capacities of seepage within the slope of central cell is:

17 $$Q_{out\_s} = v_{s} \cdot A_{s} \cdot \Delta t = K_{s} \cdot \sin \alpha \cdot d \cdot z_{s} \cdot \Delta t$$

where, *A*_*s*_ is the cross-sectional area of the water flow within the slope of central cell.

##### Runoff flow

The total runoff obtained by neighboring cells is the total water allocated by the central cell. However, within the CA model where a unified time step ∆*t* is employed, the length of the time step determines the specific flow from the central cell to the neighboring cells. When ∆*t* > T, neighboring cells can obtain all the allocated water *Q*_p_ in that direction. When ∆*t* < T, the allocated water needs to be reduced proportionally. The time duration *T*, for rainwater to flow from the central cell to its neighboring cell, along with the corresponding actual flow amount, *Q*, can be calculated as follows:

18 $$T = \frac{L}{v}$$

19 $$Q = \left\{ {\begin{array}{*{20}c} {Q_{p} \times \Delta t} & {\Delta t > T} \\ {Q_{p} \times \frac{\Delta t}{T}} & {\Delta t \le T} \\ \end{array} } \right.$$

where, *L* is the slope length between the central cell and its neighboring cell. Assuming the width of the cell is *d*. If the neighboring cell is situated in any of the four cardinal directions—up, down, left, and right—relative to the central cell, *L* = *d*, if it is located in the diagonal direction, *L* = $\sqrt{2}d$, *V* is *v*_*sf*_ or *v*_*s*_; *Q*_*p*_ is *Q*_*out_sf*_ or *Q*_*out_s*_.

#### Rainwater change

In the CA model, all cells exhibit temporal and spatial discreteness, with each cell allocates rainwater to its neighboring cells while simultaneously receiving rainwater from them. Therefore, after each time step, the change value of surface rainwater, denoted as *Q*_*sf*_, or the change value of inner rainwater, denoted as *Q*_*ud*_, of the cells can be expressed as follows:

20 $$Q_{t} = Q_{t - 1} + \sum\limits_{i = 1}^{8} {(Q_{in}^{i} )_{t} - (Q_{out} } )_{t}$$

where, *Q*_*t*_ is *Q*_*sf*_ or *Q*_*ud*_ of the central cell at time *t*, Qt-1 is *Q*_*sf*_ or *Q*_*ud*_ of the central cell at time *t*-1, ${({Q}_{in}^{i})}_{t}$ is the slope surface rainwater inflow ${({Q}_{in-sf}^{i})}_{t}$ or slope interior rainwater inflow ${({Q}_{in-ud}^{i})}_{t}$ of neighboring cell *i* at time *t*, (*Q*_*out*_)_*t*_ is the slope surface rainwater outflow (*Q*_*ou-sft*_)_*t*_ or slope interior rainwater outflow (*Q*_*out-ud*_)_*t*_ of central cell at time *t*.

#### Calculation process

**Figure 7:**
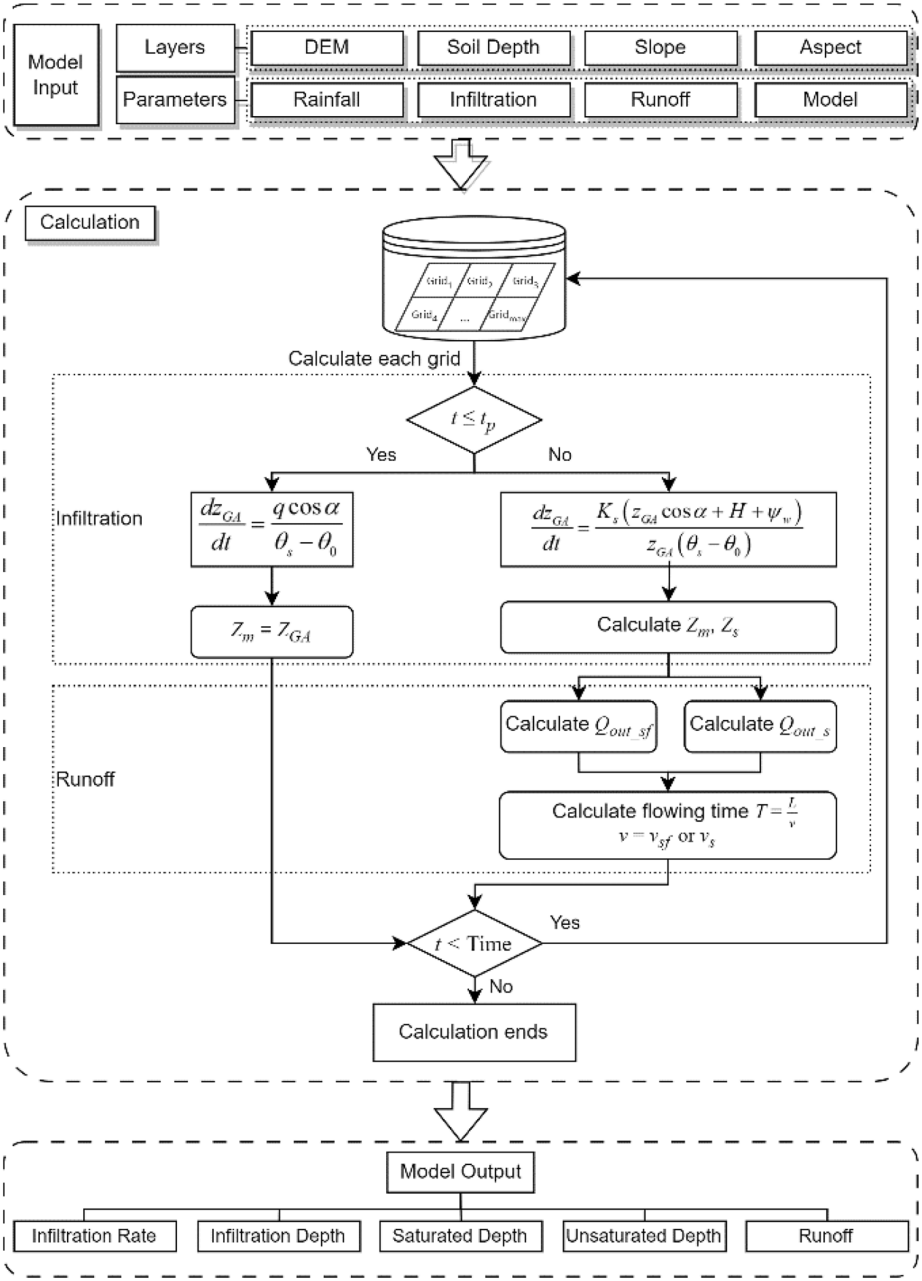
Flowchart of the rainwater movement model procedure.

Based on the Grid data format, we have employed Python programming language to develop a GIS-and-CA-based slope rainwater movement model. The flowchart of the model procedure is shown in Fig. 7, and the detailed steps are as follows:

1. Data importation. The main data includes basic raster layers such as Digital Elevation Model (DEM), soil thickness, groundwater depth, as well as topographic raster layers such as slope, aspect, and flow direction etc.
2. Parameter input. The hydraulic parameters mainly including saturated moisture content, initial moisture content, saturated permeability coefficient, matrix suction at the wetting front. The rainfall parameters encompass rainfall duration and intensity.
3. Infiltration calculation. According to the previously proposed formula, the fourth order Runge–Kutta method is used to calculate the infiltration depth and ponding of each cell in order from left to right, top to bottom. Subsequently, the calculated results are saved in the corresponding new raster layer.
4. Runoff calculation. Start the calculation from the top-left corner towards bottom-right corner of the entire grid according to the ponding result from step 3. For those ponding cells, runoff calculations are executed under established runoff rules. During the calculation process, if runoff is directed towards the left or upward direction, the respective cell is marked. Consequently, a repeated runoff calculation is performed specifically for these marked cells. Store the calculated results in new raster layer.
5. Repeat steps 3 and 4 until the rainfall process ends.
6. Results storage. Following the specified output requirements, the calculated results, such as infiltration depth, runoff and others, obtained from each cell, are individually saved within a new raster layer.

## Model validation

**Figure 8:**
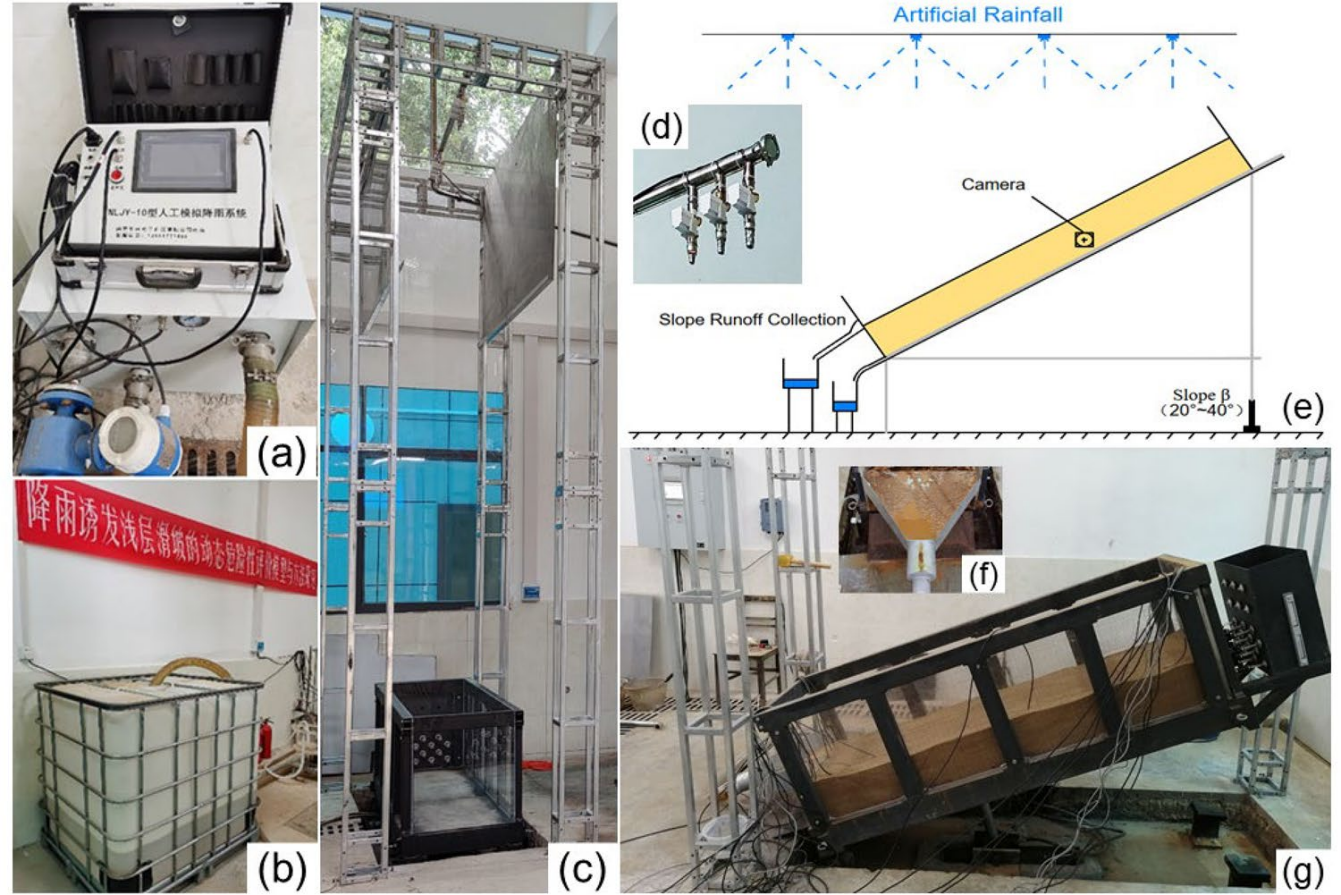
Physical model test equipment for simulating slope rainfall infiltration and runoff. (**a**) NLJY-10 artificial rainfall simulator, (**b**) water tank, (**c**) front view of test equipment, (**d**)** a set of **nozzles **with apertures of 4.5mm, 1.0mm, and 2.0mm**, (**e**) Schematic diagram of experimental model, (**f**) Rainwater runoff collection device, (**g**) side view of the runoff flume.

**Figure 9:**
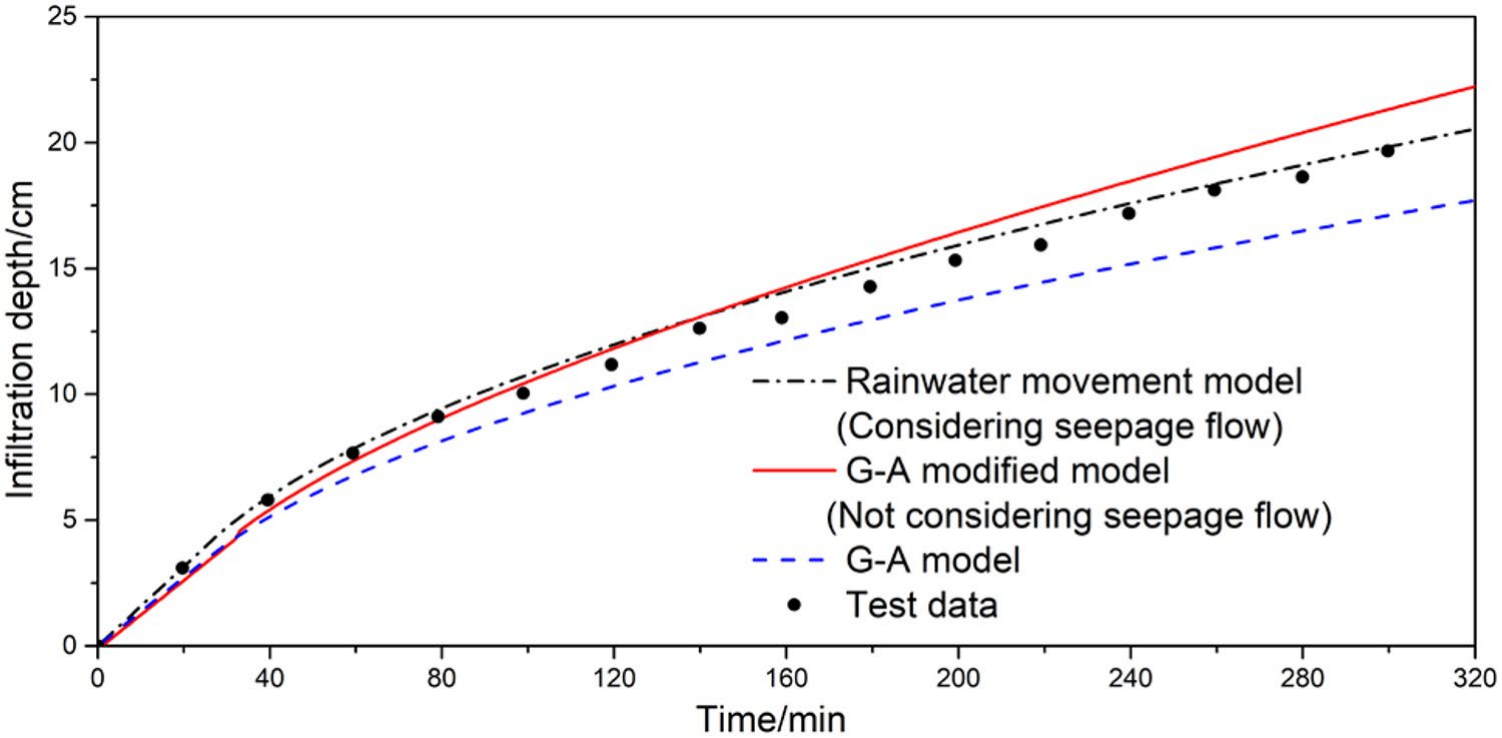
Time variation curve of wetting front in slope rainfall infiltration test.

**Figure 10:**
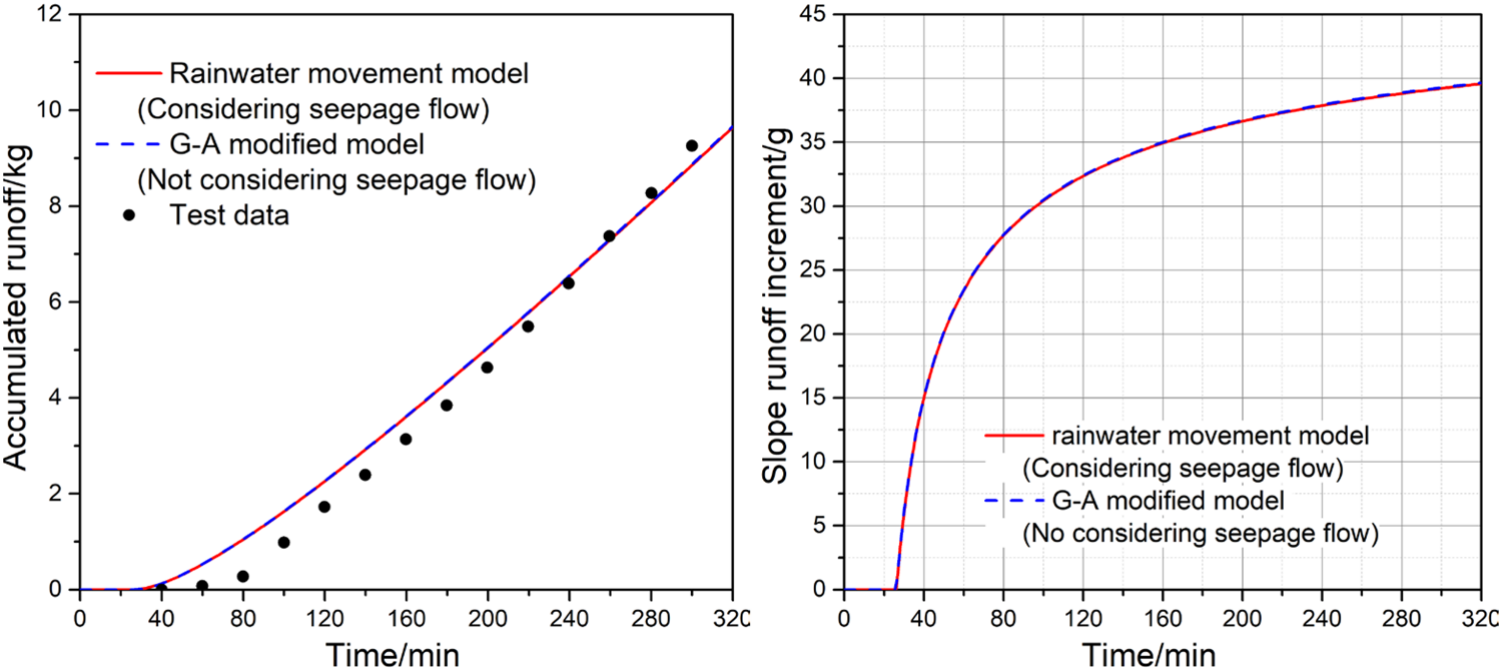
Time variation curve of runoff in slope rainfall infiltration test.

We conducted rainfall infiltration and runoff experiments to verify the reliability of the method proposed in this article for simulating the rainfall infiltration and runoff processes on unsaturated soil slopes. The experiment equipment comprised a runoff flume, an NLJY-10 artificial rainfall simulator, and a rainwater collection device (Fig. 8). The size of the flume was 2 m × 0.5 m × 0.5 m(*l* × *w* × *h*), and the experimental slope was 20°. The NLJY-10 artificial rainfall simulator comprised a rainfall controller, catheter, and sprinklers The sprinklers device contained 2 sets of nozzles with apertures of 4.5mm, 1.0mm, and 2.0mm and was placed approximately 3 m from the ground, which was directly above the runoff flume. The simulator could simulate rainfall with the intensity of 10-150 mm/h, and raindrop diameters ranging from 0.3 mm to 4 mm. The rainfall uniformity was 85.4%, which met the requirements of more than 80%. The soils depth was 0.2 m, the soil porosity was 0.6, the rainfall intensity was 30.0 mm/h, the saturated permeability coefficient was 3.26 × 10^–5^ cm/s, the initial volume moisture content was 0.12, the saturated volume moisture content was 0.48. Other parameters are shown in Table 1. We used the G-A model, the G-A modified model, and the rainwater movement model to compare. The calculated infiltration depth and slope runoff at different times are shown in Fig. 9 and Fig. 10.

**Table 1:** Parameters related to simulation experiments of rainfall infiltration and runoff on slopes.

*a*	*b*	*Ψ*	*α*	*m*	*n*
− 0.0001	0.93	1.5	0.073	0.442	1.79

Figure 9 illustrates that the cumulative infiltration depth calculated by the original G-A model consistently falls short of the experimental observations. Initially, at 20 min, the difference stands at − 0.38162. However, over time, this discrepancy widens, reaching − 2.55408 by 300 min. In contrast, the G-A modified model underestimates the infiltration depth during the first 80 min but then exceeds the experimental values, resulting in a difference of 1.65545 at 300 min. Conversely, the rainwater movement model consistently overestimates the infiltration depth, with a difference of 0.17 at 300 min.

To accurately assess the degree of deviation between the simulated and observed values, we calculated the Root-Mean-Square Deviation (RMSD). A lower RMSD indicates a closer approximation of the simulated infiltration depth to the experimental observations. Our analysis reveals that the rainwater movement model boasts the lowest RMSD value of 0.567, suggesting its results are the closest to the experimental observations. In contrast, the G-A model exhibits the highest RMSD value of 1.477, indicating a relatively lower degree of accuracy in its calculations. The G-A modified model falls somewhere in the middle, with an RMSD value of 1.061. Overall, the rainwater movement model emerges as the most accurate in simulating infiltration depth.

From Fig. 10, it is evident that the calculation results exhibit a high degree of consistency, regardless of whether slope infiltration is considered or not. Compared to the actual measurements, the model used in this study initially overestimates the runoff volume but gradually approaches the observed data. The simulated runoff generation time is approximately 30 min, whereas the actual observed time is about 40 min. Furthermore, the observed runoff process demonstrates a gradual curved increase, whereas the calculated values exhibit a more uniform linear growth pattern. By comparing the runoff increments per unit time, we observe a fundamental agreement between the two. As time progresses, the slope of the runoff increment curve gradually decreases, indicating that the rainfall runoff increment increases with time until it reaches a steady value. This trend aligns with the temporal variation of rainfall infiltration rate. Over time, the amount of rainfall infiltration decreases, while the slope runoff gradually increases until both reach their respective constant values.

In summary, the calculation results of the rainwater movement model closely reflect the actual patterns of rainfall infiltration and runoff on slopes, making it a viable simulation model for predicting rainwater movement on slopes.

## Case Study

### Study area background

**Figure 11:**
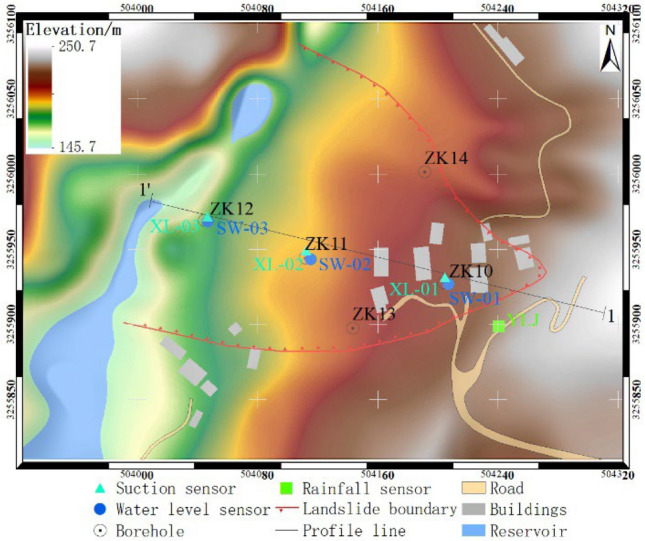
Plane zoning map of the Chengxiyu landslide.

**Figure 12:**
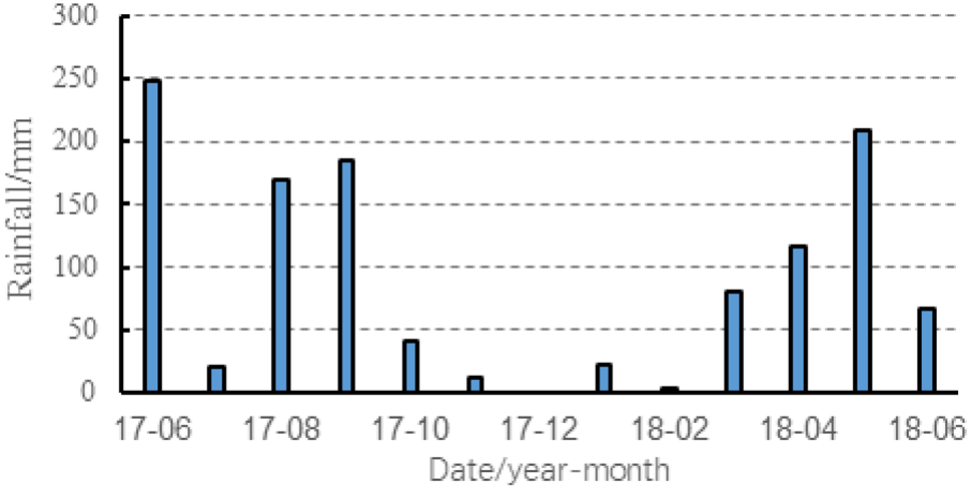
Monthly rainfall of Chenxiyu landslide from 2017/6 to 2018/6.

**Figure 13:**
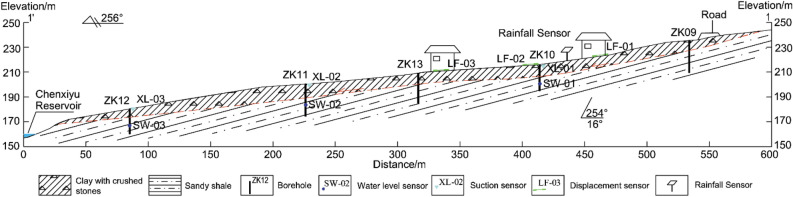
Slope profile map.

The study area is situated in Chenxiyu Village, Cili County, Zhangjiajie City, China. The slope exhibits a tongue-like shape and possesses a relative height difference of approximately 60 m. The thickness of the Quaternary deposit on the slope is about 5 m, as shown in Fig. 11. During the period from 2017 to 2018, the total annual rainfall in the slope area was about 1175 mm. Most of this rainfall was concentrated during the months of May to August, With June 2017 recording the highest monthly total of 248.2 mm. The maximum daily rainfall, measuring 70 mm, occurred on August 12, 2017, as shown in Fig. 12. Due to the influence of rainfall and reservoir water level fluctuations, the slope has undergone varying degrees of deformation^34^. To analyze the movement patterns of rainwater inside the slope and consequently obtain a more profound comprehension of its deformation mechanism, laboratory tests and on-site monitoring was conducted on the slope. The plane and the profile of the monitoring sensors' location are shown in Fig. 11 and Fig. 13.

### Model parameters and calculation conditions

**Figure 14:**
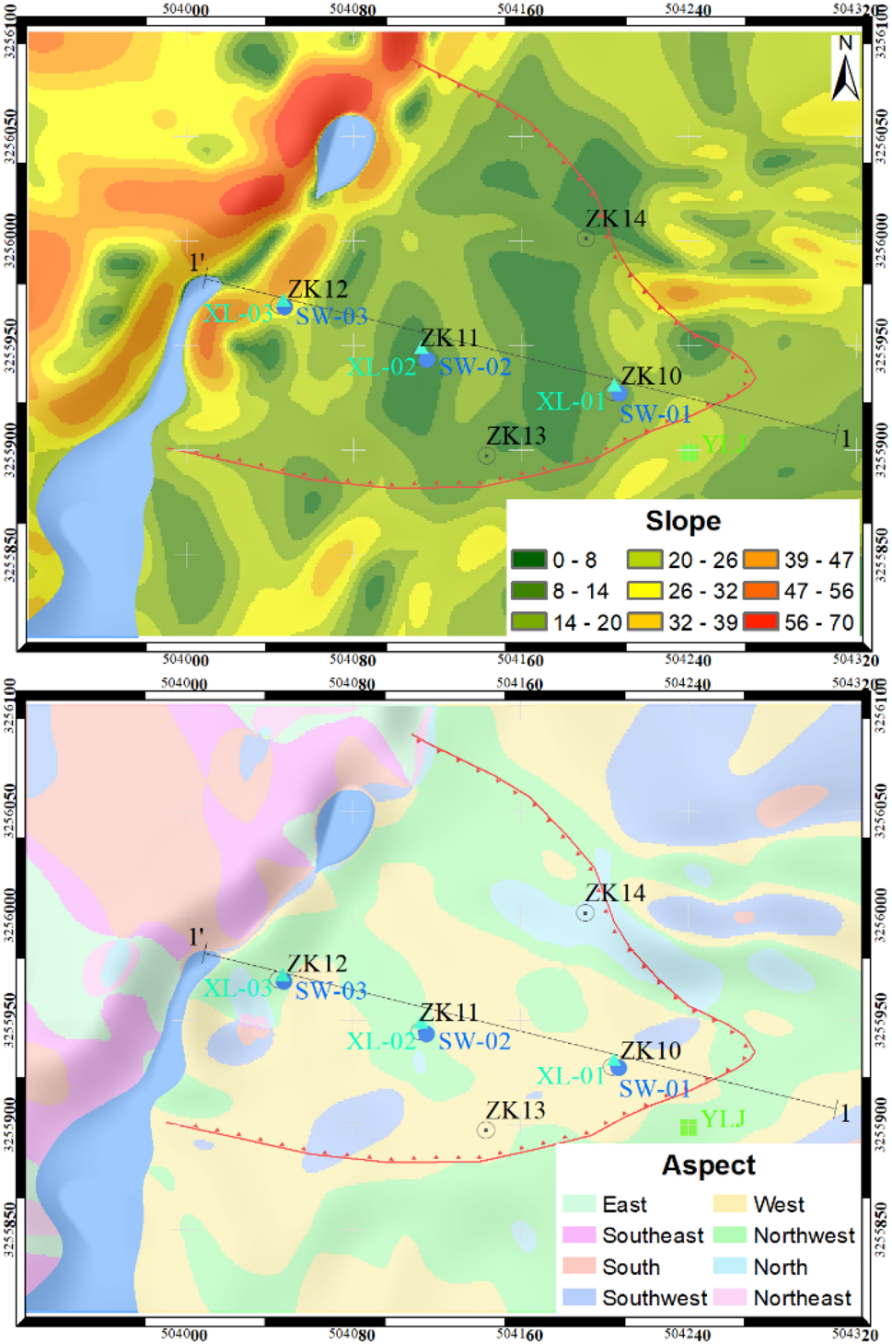
Layer parameters of study area.

The Chenxiyu slope underwent rasterization using a grid size of 1 m × 1 m, dividing it into a total of 291 × 400 cells. The slope and flow direction within the study area are illustrated in Fig. 14.

#### Soil parameters

**Figure 15:**
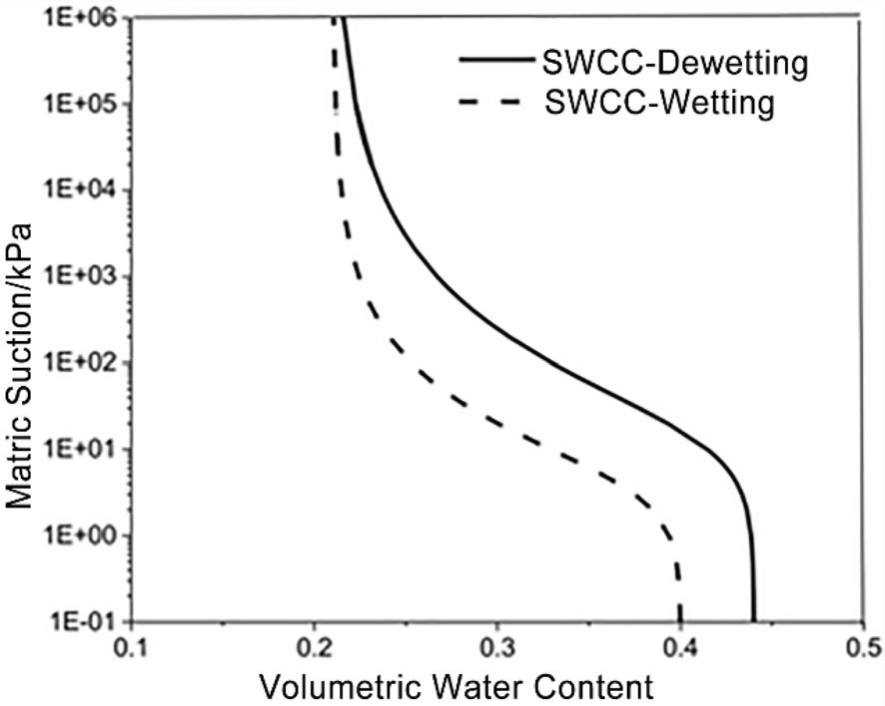
Soil and water characteristic curves (SWCC).

The soil on the Chenxiyu slope is mainly composed of silty clay. Laboratory tests were conducted using soil samples collected from various borehole along the profile, the saturated permeability coefficient of the soil was measured as 3.3 × 10^-6^m/s. Additionally, the soil water characteristic curve is shown in Fig. 15, and the soil parameters are shown in Table 2.

**Table 2:** Soil property parameters of Chenxiyu slope.

*a*	*b*	*Ψ*	*θ*_*0*_	*θ*_*s*_
− 0.00005	0.95	200.0	0.25	0.40

#### Calculation conditions

**Figure 16:**
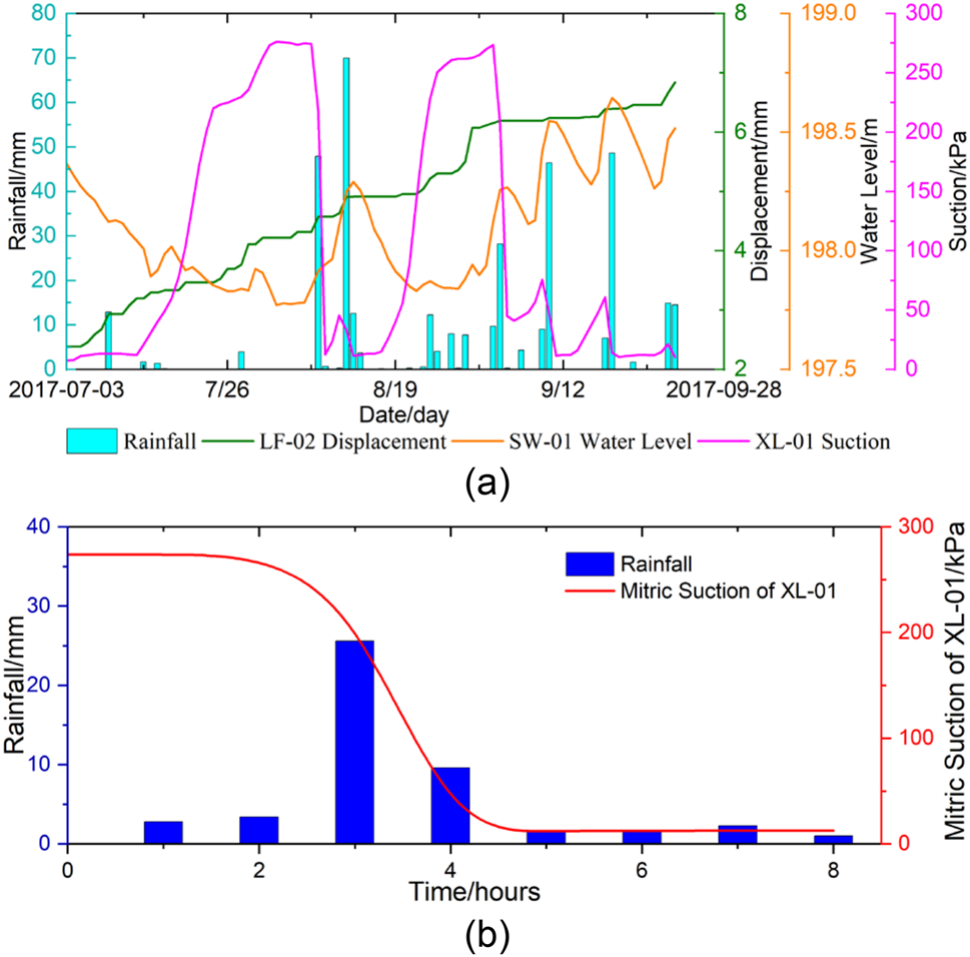
Monitoring curve of rainfall, cumulative displacement, groundwater level and Matrix suction in ZK10.

In June 2017, automatic monitoring devices such as rain gauge, matrix suction, water level, and crack displacement sensors were installed on the Chenxiyu slope^34^. The observed data of groundwater level (SW-01), rainfall, matrix suction (XL-01) and displacement sensors (LF-02) in ZK10 from July to September 2017 are shown in Fig. 16a. XL-01 was located at a depth of 65 cm, approximately 90 cm away from SW-01, LF-02 was placed across the crack on the surface of the slope.

A simulation study was conducted on a rainfall process during August 8, 2017, with a rainfall duration of 480 min and the rainfall intensity are shown in Fig. 16b.

## Results analysis

**Figure 17:**
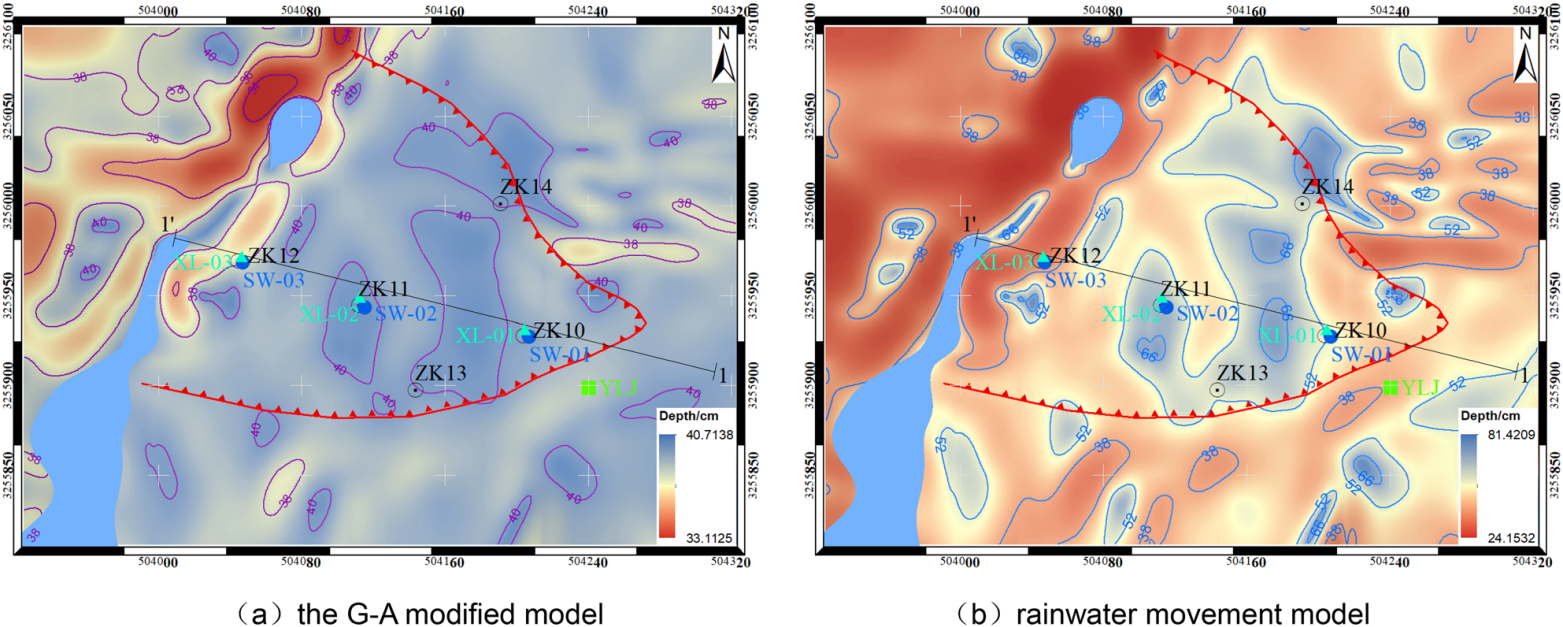
Distribution map of wetting front depth on Chenxiyu slope.

**Figure 18:**
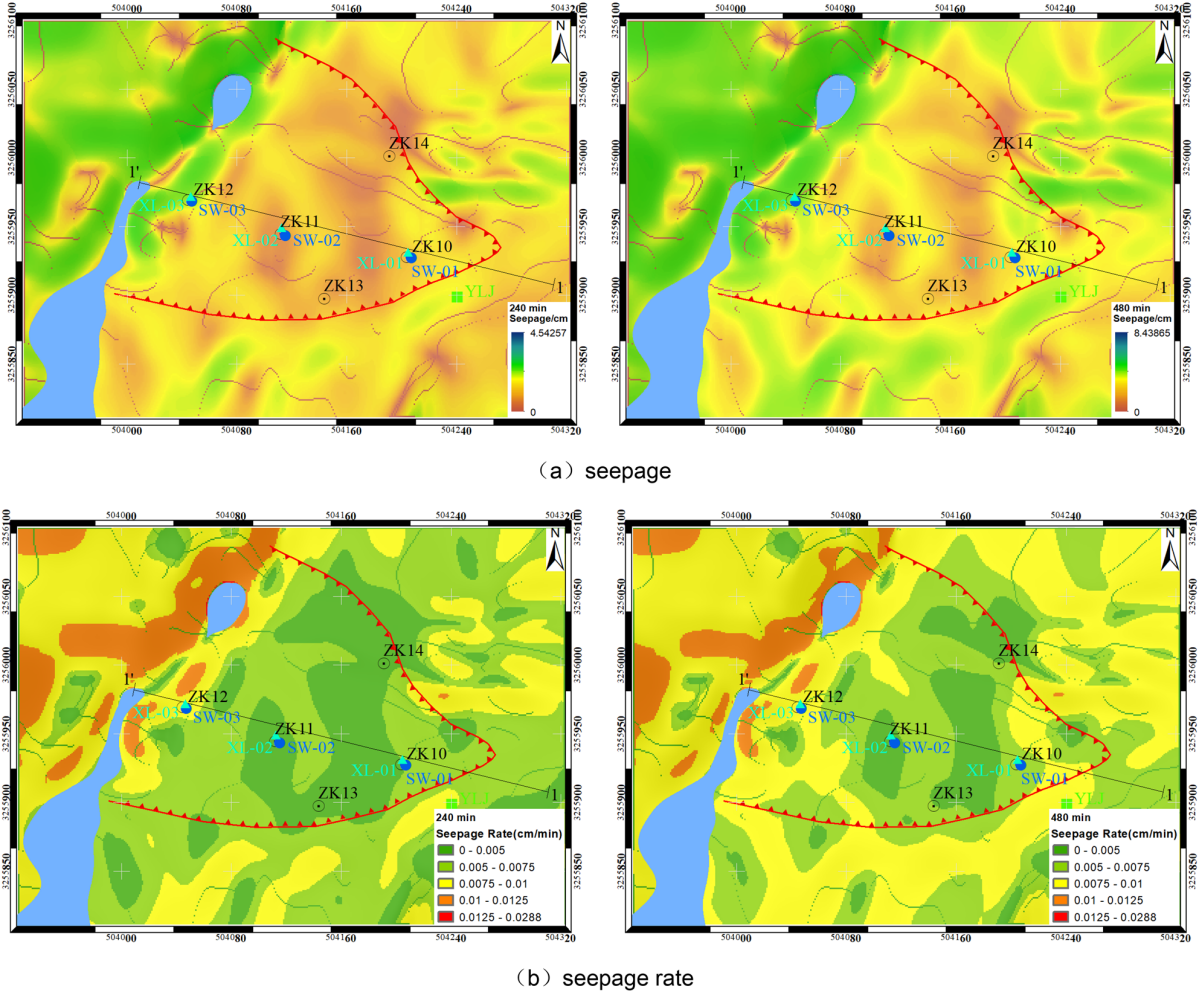
Calculation results of seepage at different times on the slope of Chenxiyu.

**Figure 19:**
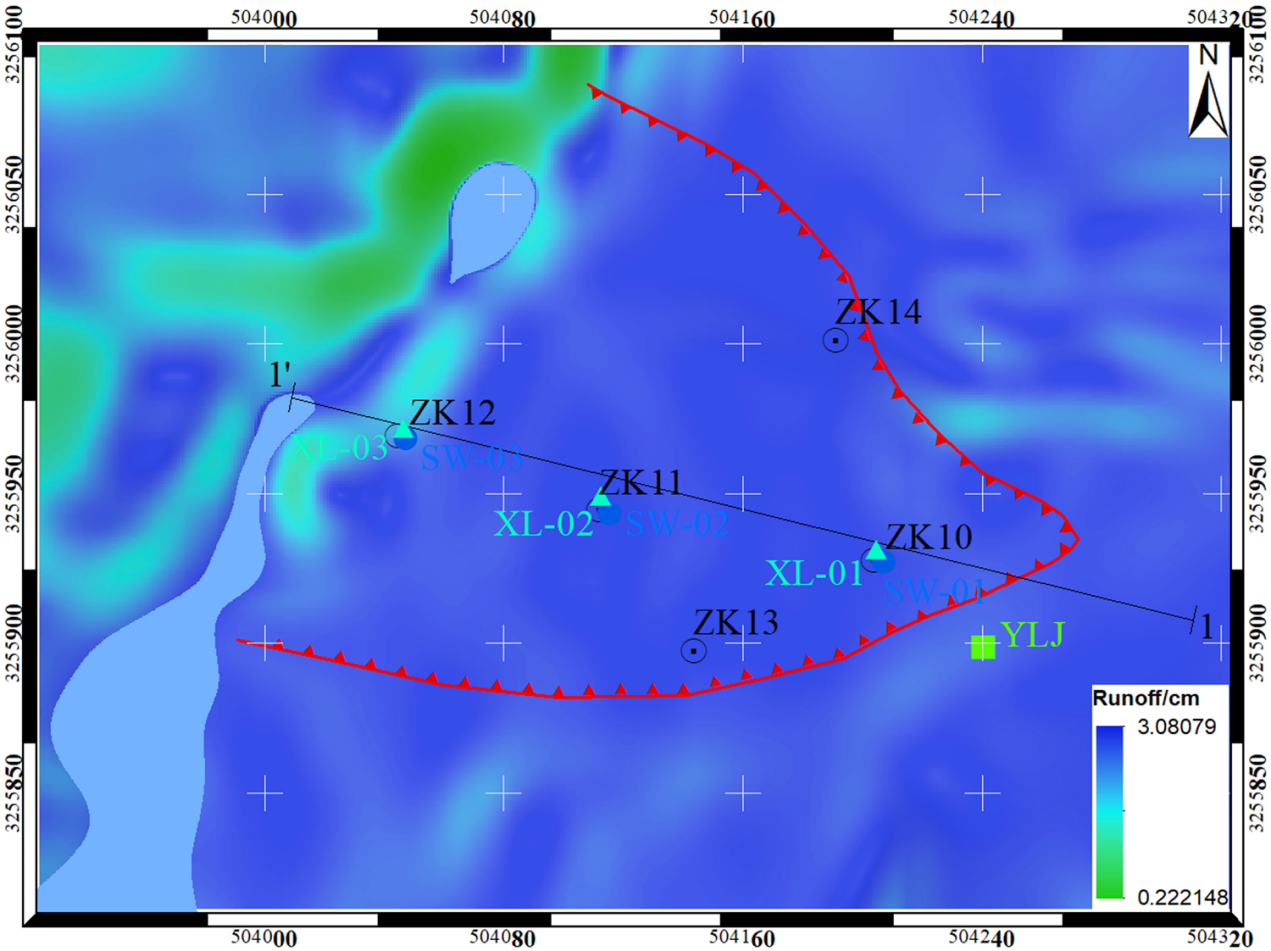
Calculation results of cumulative runoff depth on Chenxiyu slope.

Based on the simulation parameters and calculation conditions mentioned above, the rainfall process of the Chenxiyu slope was simulated and analyzed using the rainwater movement model proposed in this paper. The calculation results of the wetting front depth on the Chenxiyu slope are shown in Fig. 17. The calculation results of seepage at different times are shown in Fig. 18, and the calculation results of cumulative runoff are shown in Fig. 19.

According to Fig. 17, the average infiltration depth calculated by the G-A modified model in the study area is approximately 36.91 cm. Specifically, the infiltration depth near the Chenxiyu slope is about 40 cm, while the minimum infiltration depth of approximately 33.11 cm is observed in the northern part of the study area opposite the slope. This region is generally steeper, with an average slope of approximately 60°. The steeper the slope, the less rainwater is distributed across the slope surface, resulting in reduced infiltration.

On the other hand, the results from the rainwater movement model reveal that the average infiltration depth in the study area is 52.79 cm. Notably, the infiltration depth at the steep slope in the northern part of the study area is approximately 24.15 cm. This indicates that the steeper the slope, the greater the seepage flow and the shallower the infiltration depth.

Figure 18 illustrates the seepage flow (a) and seepage velocity (b) at various time points calculated using the rainwater movement model. The seepage velocities are categorized into five levels: very low (< 0.005 cm/min), low (0.005–0.0075 cm/min), medium (0.0075–0.01 cm/min), high (0.01–0.0125 cm/min), and very high (> 0.0125 cm/min). The results reveal that the average seepage flows at 240 min and 480 min are 2.27 cm and 4.22 cm, respectively, with corresponding average seepage velocities of 0.0094 cm/min and 0.0087 cm/min. This indicates a gradual decrease in the average seepage capacity of the study area over time. Furthermore, the classification of seepage velocities demonstrates that during the entire rainfall process, the steep slopes exhibit high runoff velocities. Conversely, the proportions of areas with low and very low runoff velocities decrease by 5.1% and 11.5%, respectively. Meanwhile, the medium runoff velocity areas increase from 28.4% to 44.0%. Notably, the seepage capacity of the Chenxiyu slope appears to strengthen over time.

In Fig. 19, the average depth of cumulative runoff on the Chenxiyu slope is about 1.65 cm and the depth of surface runoff per minute is about 0.034 mm. Therefore, during the entire rainfall process, the depth of cumulative runoff per minute is relatively small.

## Discussion

In indoor tests, we observed that the measured runoff process was relatively smooth, while the calculated runoff curve was steeper. The reason for this discrepancy is that the simulation calculation is more accurate. Once there is ponding, the runoff calculation will be immediately performed and the runoff volume will be obtained. In contrast, the measured results may be affected by the complexity of the surface runoff on the slope, resulting in the collection of rainwater on the front edge of the slope only at a later time after ponding. This phenomenon is basically consistent with the E1-3 runoff experimental simulation results conducted by Tang et al.^25^.

It is worth noting that the model in this study only modified the infiltration depth, while the infiltration rate of the model remained consistent with that of the original G-A Model. Therefore, by comparing the increase in runoff per unit time, we found that the two were basically consistent. This finding provides important information about the performance of the model and provides direction for future model improvements.

In the case study, the infiltration depth calculated by the Rainwater Movement Model was generally larger than that of the G-A Modified Model. Specifically, the infiltration depth in the middle of the Chenxiyu slope was deeper than that in the upper and lower parts. Combining this observation with the slope gradient map, it becomes evident that there is a strong negative correlation between the infiltration capacity of the slope and its gradient. The flatter the slope, the deeper the infiltration depth.

Near the location of ZK10, the infiltration depth calculated by Rainwater Movement Model was approximately 52 cm, whereas G-A Modified Model estimated it to be around 39 cm. When comparing this data with the monitoring curve of the matric suction meter XL-01 shown in Fig. 16b, it is notable that five hours after the rainfall, XL-01 reached its minimum value, indicating that the soil at the depth of 65 cm (where XL-01 is located) was in a saturated state. Given that the rainfall intensity after 5 h was minimal, its impact on subsequent infiltration was also negligible. By comparing the calculation results of both models, it is apparent that the infiltration depth estimated by Rainwater Movement Model is closer to the monitoring results obtained from XL-01.

After analyzing the seepage calculation results, it becomes evident that there has been a notable increase in the overall seepage velocity within the Chenxiyu landslide area. This increase results in the formation of a significant infiltration force within the slope, which subsequently has a considerable impact on the stability of the landslide. Consequently, this infiltration force triggers creep deformation, leading to a discernible upward trend in the monitored displacement data of the landslide. Specifically, Fig. 16a illustrates an approximate increase of 5 mm in the cumulative displacement.

The cumulative runoff exhibits a noticeable pattern in the study area, being more prominent on the Chenxiyu slope and less so on the northwestern slope, where the gradient is markedly steeper than that of the southeastern slope. Case studies have revealed that steeper slopes typically receive lesser amounts of rainfall per unit area, leading to a delayed initial runoff time and consequently, reduced runoff generation. This observation aligns with the findings of Lei et al.^35^, who conducted physical simulation experiments and discovered that the runoff initiation time decreases as the slope gradient increases.

Overall, this paper was focus on the introduction and the application of the model, which was only tested in limited experiment. Based on the results of the experiment and case, there is promising potential for successfully applying this model to other spatial scales and under more complex rainfall conditions.

## Conclusion

A GIS&CA-based rainwater movement model in slope scale has been developed to simulate rainfall infiltration, surface runoff and saturated seepage at different rainfall times. This model uses grid data format and integrates with GIS, enabling accurate scenario simulation calculations and convenient results display. The model modifies the G-A infiltration model and improves the rules of runoff generation and convergence within and on the slope, which can effectively simulate the spatiotemporal changes of rainfall infiltration and runoff. It can also be used as an effective tool for analyzing characteristics of underground and surface water movement and assessing rainfall-induced landslide stability in different spatial scales for event-based simulations.

The accuracy of the model was verified through laboratory experiments. The results indicate that the infiltration calculation results of the model are closer to the experimental values, and there is a certain deviation between the runoff calculation results and the experimental values. Application at Chenxiyu slope showed that it had better performance compared to G-A modified model in predicting the infiltration depth and runoff. The rainfall movement model can be used to describe the spatial distribution and temporal variation of infiltration and runoff processes.

The process of rainwater movement on slopes is a complex system that includes multiple influencing factors. In this study, only factors such as rainfall, soil, and terrain were considered, while some factors such as vegetation, surface evaporation, and temperature were ignored. If more cell states and transition rules are added, it will undoubtedly improve the accuracy of the model and make the results more ideal. In addition, the entire calculation of the region used the same parameter, which differs greatly from the actual situation. In further study, the spatial differences of soil parameters should be considered, and different soil parameters should be assigned to different regions for calculation, and the coupling method of rainwater movement process between regions should be studied.

## Data Availability

The datasets generated during and/or analyzed during the current study are available from the corresponding author on reasonable request.

## References

[1] Richard-M I (2000). Landslide triggering by rain infiltration[J]. Water Resour. Res..

[2] Marianna P, Raffaele P, Marco-Valerio N (2015). In situ monitoring of the groundwater field in an unsaturated pyroclastic slope for slope stability evaluation[J]. Landslides.

[3] Sabatino C, Maria DS (2015). Large-area analysis of soil erosion and landslides induced by rainfall: A case of unsaturated shallow deposits[J]. J. Mountain Sci..

[4] Mohsen ET, Behzad AA (2019). A modeling platform for landslide stability: A hydrological approach[Z]. Water.

[5] Heber G, Ampt G-A (1911). Studies on soil physics: Part I—the flow of air and water through soils[J]. J. Agricult. Sci..

[6] Kostiakov A-N. On the dynamics of the coefficient of water‐percolation in soils and on the necessity for studying it from a dynamic point of view for purposes of amelioration[C]. Sixth Comm. Int. Soil Sci. SocMoscow, Moscow, Part A,17–21. (1932).

[7] Horton Robert-E. An Approach Toward a Physical Interpretation of Infiltration Capacity[C]: Madison, (1940).

[8] Philip JR (1955). Numerical solution of equations of the diffusion type with diffusivity concentration-dependent[J]. Trans. Faraday Soc...

[9] Renato M, Corrado C, Carla S (2018). Rainfall infiltration modeling: A review[J]. Water.

[10] Shakir A, Adlul I (2018). Solution to green-ampt infiltration model using a two-step curve-fitting approach[J]. Environ. Earth Sci..

[11] Ravindra-V K, Bhabagrahi S (2011). Green-ampt infiltration models for varied field conditions: A revisit[J]. Water Resour. Manag...

[12] Li Li, Dongsheng Z, Bo Ni (2022). Study on the stability analysis of rainfall slope based on g-a model considering moisture content[J]. Sci. Rep..

[13] Shaohong Li, Peng C, Ping C (2022). Modified green-ampt model considering vegetation root effect and redistribution characteristics for slope stability analysis[J]. Water Resour. Manag..

[14] Zi-zhen L, Zhi-xin Y, Zhan-hong Q (2020). Stability analysis of an unsaturated soil slope considering rainfall infiltration based on the green-ampt model[J]. J. Mountain Sci..

[15] Hong-Kang Ji, Majid M, Sai-Hin L (2023). The robustness of conceptual rainfall-runoff modelling under climate variability—A review[J]. J. Hydrol..

[16] Teng J, Jakeman A-J, Vaze J (2017). Flood inundation modelling: A review of methods, recent advances and uncertainty analysis[J]. Envirn. Modell. Softw..

[17] Beven K, Calver A, Morris E-M. The Institute of Hydrology Distributed Model[R]. Institute of Hydrology Report No. 98, (1987).

[18] Bathurst J-C, Wicks J-M, O'Connell P-E (1995). The She/Shgesed Basin Scale Water Flow and Sediment Transport Modelling System[M].

[19] Keith B (1997). Topmodel; A critique[J]. Hydrol. Process..

[20] Maidment D-R. Arc Hydro: Gis for Water Resources[M]: ESRI Press, (2002).

[21] Neitsch Susan-L, Arnold Jeffrey-G, Kiniry Jim-R, et al. Soil and Water Assessment Tool Theoretical Documentation Version 2009[Z]. Texas Water Resources Institute, (2011).

[22] Scharffenberg W, Estats Units-D'Amèrica.-Army.-Corps. Hydrologic Modeling System Hec-Hms: User's Manual[M]. (US Army Corps of Engineers, 2016).

[23] Nguyen-Thi A (2016). The application of cellular automata to investigate runoff on surface of complex topography under different rainfall scenario[j]. Int. J. GEOMATE.

[24] Qi S, Dion W, Longbin H (2015). Runca: A cellular automata model for simulating surface runoff at different scales[J]. J. Hydrol..

[25] Shengqiang T, Dongli S, Hongde W (2022). An improved cellular automata model for soil erosion in coastal areas based on discrete physical variables[J]. Eur. J. Soil Sci..

[26] Javier-Eugenio VB, Jérôme-Leboeuf P, Juan-De-Dios BS (2022). Wildfire effect on forest rainfall infiltration and runoff: A cellular automata-based simulation[J]. Int. J. Hydrol..

[27] Shuang Y, Chen Nengcheng Du, Wenying,  (2021). A Cellular automata based rainfall-runoff model for urban inundation analysis under different land uses[J]. Water Resour Manag..

[28] Li C, Michael-H Y (2006). Green-ampt infiltration model for sloping surfaces[J]. Water Resour. Res..

[29] Russell-G M, Curtis-L L (1973). Modeling infiltration during a steady rain[J]. Water Resour. Res..

[30] Hu Hai-jun, Li Bo-peng, Tian Kan-liang, et al. Simulation of Water Movement in Unsaturated Remolded Loess Under Ponding Infiltration and Rainfall Infiltration[J]. *Journal of Tongji University (Natural Science)*, **47**(11), 1565–1573 (2019).

[31] Zhen-yang P, Huang Jie-sheng Wu, Jing-wei,  (2012). Improvement of green ampt model based on layered hypothesis[J]. Adv. Water Sci..

[32] Nándor F, Renáta S, Tomas O (2011). Evaluation method dependency of measured saturated hydraulic conductivity[J]. Geoderma.

[33] Susan-K J, Julia-O D (1988). Extracting topographic structure from digital elevation data for geographic information system analysis[J]. Photogrammetr. Eng. Remote Sens..

[34] Liu Lei Xu, Yong LY (2019). A study of deformation mechanism and stability evaluation of the Chenxiyu landslide in western Hunan[J]. Hydrogeol. Eng. Geol..

[35] Wenkai L, Hongyuan D, Pan C (2020). Study on runoff and infiltration for expansive soil slopes in simulated rainfall[J]. Water.

